# Endogenous sulfur dioxide alleviates collagen remodeling via inhibiting TGF-β/Smad pathway in vascular smooth muscle cells

**DOI:** 10.1038/srep19503

**Published:** 2016-01-14

**Authors:** Yaqian Huang, Zhizhou Shen, Qinghua Chen, Pan Huang, Heng Zhang, Shuxu Du, Bin Geng, Chunyu Zhang, Kun Li, Chaoshu Tang, Junbao Du, Hongfang Jin

**Affiliations:** 1 Department of Pediatrics, Peking University First Hospital, Beijing 100034, P. R. China; 2 Department of Endocrinology, Beijing Chao-Yang Hospital, Capital Medical University, Beijing 100020, P. R. China; 3 Department of Pediatrics, Capital Medical University Shijitan Hospital, Beijing 100038, P. R. China; 4 Department of Physiology and Pathophysiology, Peking University Health Science Centre, Beijing 100191, P. R. China; 5 Key Laboratory of Green Chemistry and Technology, Ministry of Education, College of Chemistry, Sichuan University, Chengdu, 610064, P. R. China; 6 Key Laboratory of Molecular Cardiology, Ministry of Education, Beijing 100191, P. R. China

## Abstract

The study was designed to investigate the role of endogenous sulfur dioxide (SO_2_) in collagen remodeling and its mechanisms in vascular smooth muscle cells (VSMCs). Overexpression of endogenous SO_2_ synthase aspartate aminotransferase (AAT) 1 or 2 increased SO_2_ levels and inhibited collagen I and III expressions induced by transforming growth factor (TGF)-β1 in VSMCs. In contrast, AAT1 or AAT2 knockdown induced a severe collagen deposition in TGF-β1-treated VSMCs. Furthermore, AAT1 or AAT2 overexpression suppressed procollagen I and III mRNA, upregulated matrix metalloproteinase (MMP)-13 expression, downregulated tissue inhibitors of MMP-1 level, and vice versa. Mechanistically, AAT1 or AAT2 overexpression inhibited phosphorylation of type I TGF-β receptor (TβRI) and Smad2/3 in TGF-β1-stimulated VSMCs. Whereas SB431542, an inhibitor of TGF-β1/Smad signaling pathway, attenuated excessive collagen deposition induced by AAT knockdown. Most importantly, ectopically expressing AAT or exogenous addition of 100 μM SO_2_ blocked AAT deficiency-aggravated collagen accumulation in TGF-β1-stimulatd VSMCs, while no inhibition was observed at 100 μM ethyl pyruvate. These findings indicated that endogenous SO_2_ alleviated collagen remodeling by controlling TGF-β1/TβRI/Smad2/3-mediated modulation of collagen synthesis and degradation.

Severe structural changes in vascular walls characterized by vascular collagen remodeling are central to the pathophysiology of vascular diseases such as hypertension, atherosclerosis and restenosis after coronary angioplasty^1^,^2^. Vascular collagen mainly consists of collagen I and III. Collagen I is associated with the tenacity and tensile strength of vascular wall, while collagen III is associated with the elasticity of vascular wall, both play important role in maintaining the integrity of vascular structure. Previous study indicated that vascular collagen remodeling was the consequence of an imbalance between collagen synthesis and degradation, characterized by excessive deposition of collagen, disequilibrium of collagen types (increased ratio of collagen I/III) and disorganized collagen arrangement^3^. Among them, the excessive deposition of collagen I and III in vascular wall is one of the most important factors of vascular remodeling, and is also a common consequence of many cardiovascular diseases. The synthesis and degradation of collagen in VSMC are critical to vascular remodeling^3^,^4^, but much less is known about the pathogenetic mechanism of collagen remodeling in VSMC under pathological conditions, especially the regulatory mechanism of abnormal synthesis and (or) degradation of collagen has not been fully elucidated.

More and more research has suggested that endogenous gaseous signaling molecules play important function in cardiovascular system. It was reported that the gasotransmitter hydrogen sulfide inhibited the abnormal accumulation of vascular collagen, and alleviated vascular remodeling in spontaneously hypertensive rats^5^,^6^. Sulfur dioxide (SO_2_), another gasotransmitter, shares the same substrate with hydrogen sulfide, which also can be endogenously generated from a sulfur-containing amino acid^7^. L-cysteine can be oxidized by cysteine dioxygenase to L-cysteine sulfinate, which can be transformed into β-sulfinylpyruvate by aspartate aminotransferase (AAT), and then spontaneously decomposes to pyruvate and SO_2_^8^. Study has shown that SO_2_ could inhibit hypoxic pulmonary vascular remodeling^9^. However, it’s still unclear about the regulation of endogenous SO_2_ on the collagen remodeling in VSMCs, and its possible mechanism.

Transforming growth factor-β1 (TGF-β1) is widely known as a key factor in vascular collagen remodeling, participating in the development of vascular injury in a variety of cardiovascular diseases. There are three isoforms of TGF-β, TGF-β1, TGF-β2 and TGF-β3. TGF-β1 is the major isoform of the TGF-β superfamily, can be produced by VSMCs, and regulates growth, differentiation, migration and proliferation of VSMCs as well as extracellular matrix deposition^10^,^11^. Although TGF-β1 action involve many downstream signaling pathways and cross-talk, the intracellular Smad signaling pathway is considered to play a crucial role in mediating the intracellular response to TGF-β1^12^. Activated TGF-β1 binds tightly to transmembrane type II TGF-β receptor (TβRII), a serine/threonine kinase which phosphorylates type I TGF-β receptor (TβRI). Then the phosphorylated TβRI triggers Smad2 and Smad3 phosphorylation. The phosphorylated Smad2/3 forms a complex with Smad4 and translocate from cytoplasm into the nucleus and acts as a transcription factor to enhance the transcription of collagen, matrix metalloproteinases (MMPs) and tissue inhibitors of MMPs (TIMPs). TGF-β1 directly promotes collagen synthesis and inhibits collagen degradation, thus resulting in the abnormal deposition of collagen^13^,^14^.

Based on these discoveries, we designed experiments to explore the role of endogenous SO_2_ in the TGF-β1-induced collagen remodeling in VSMCs and its possible mechanisms.

## Results

### Endogenous sulfur dioxide was associated with the inhibition of TGF-β1-induced collagen remodeling in VSMCs

**Figure 1:**
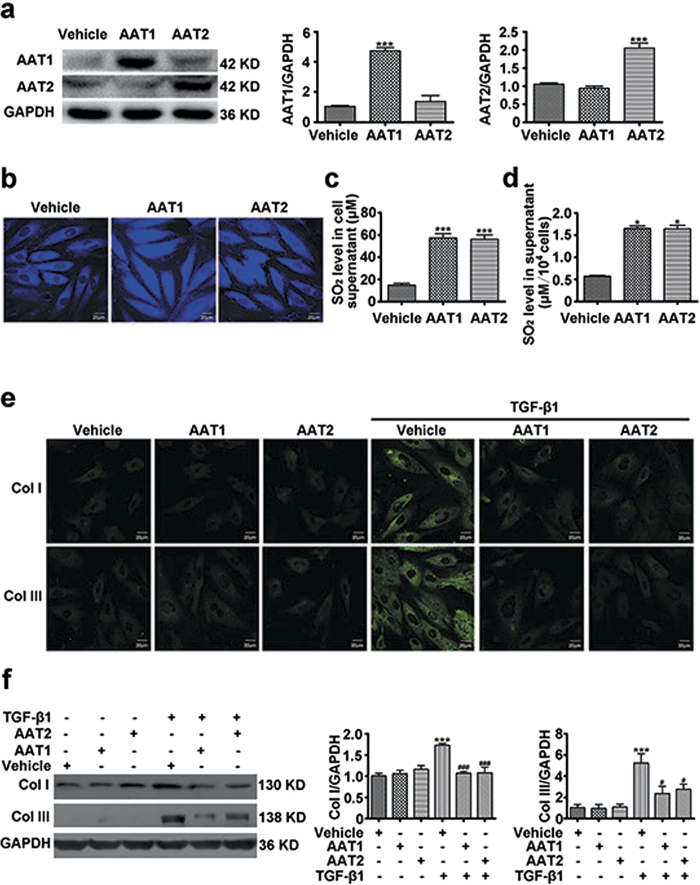
Endogenous SO_2_ overexpression inhibited TGF-β1-induced VSMC collagen remodeling. (**a**) Protein expression of AAT1 and AAT2 in VSMCs transfected with 2 μg of AAT1, AAT2 or vehicle plasmid for 48 h. (**b**) Representative fluorescent staining (blue) of endogenous SO_2_ in VSMCs transfected with AAT1, AAT2 or vehicle plasmid for 48 h. Scale bar, 20 μm. (**c**) SO_2_ content in supernatant from VSMCs transfected with AAT1, AAT2 or vehicle plasmid for 48 h. (**d**) SO_2_ content in supernatant from VSMCs transfected with AAT or vehicle plasmid was corrected with the number of cells (×10^4^). (**e**) Collagen I (Col I) and III (Col III) expression in VSMCs by confocal images. VSMCs in coverslips were transfected with AAT1, AAT2 or vehicle before TGF-β1 (10 ng/ml) stimulation for 24 h. Scale bar, 20 μm. (**f**) Representative Western blot and quantification of collagen I and III in VSMCs transfected with AAT1, AAT2 or vehicle plasmid before TGF-β1 treatment. ***P < 0.001 compared with vehicle, *P < 0.05 compared with vehicle, ^###^*P* < 0.001 or ^#^*P* < 0.05 compared with vehicle + TGF-β1 group (ANOVA). Data are represented as mean ± SD (n = 4–5).

Sulfur dioxide could be endogenously produced from L-cysteine in mammals through transamination by AAT. To investigate the effect of endogenous SO_2_ on collagen remodeling in VSMCs, we overexpressed two isozymes of AAT, AAT1 and AAT2 in VSMCs respectively. Transfection of VSMCs with AAT1 or AAT2 plasmid could significantly increase AAT1 or AAT2 protein expression as compared with vehicle (Fig. 1a). In accordance, the endogenous SO_2_ level was obviously elevated in VSMCs transfected with AAT1 or AAT2 plasmid (Fig. 1b). HPLC-FD assay also showed that much higher SO_2_ content in the supernatant from VSMCs transfected with AAT1 or AAT2 plasmid than vehicle (Fig. 1c). And the concentration of SO_2_ corrected by the number of cells was also increased significantly by AAT overexpression (Fig. 1d). Both immunofluorescence method and Western blot analysis showed that TGF-β1 could upregulated protein expression of collagen I and III in VSMCs, while AAT1 or AAT2 overexpression could significantly inhibit the TGF-β1-induced collagen I and III expression (Fig. 1e,f). Therefore, these results indicated that endogenous SO_2_ might suppress TGF-β1-induced excessive collagen deposition in VSMCs.

### Endogenous sulfur dioxide deficiency aggravated the TGF-β1-induced collagen remodeling in VSMCs

**Figure 2:**
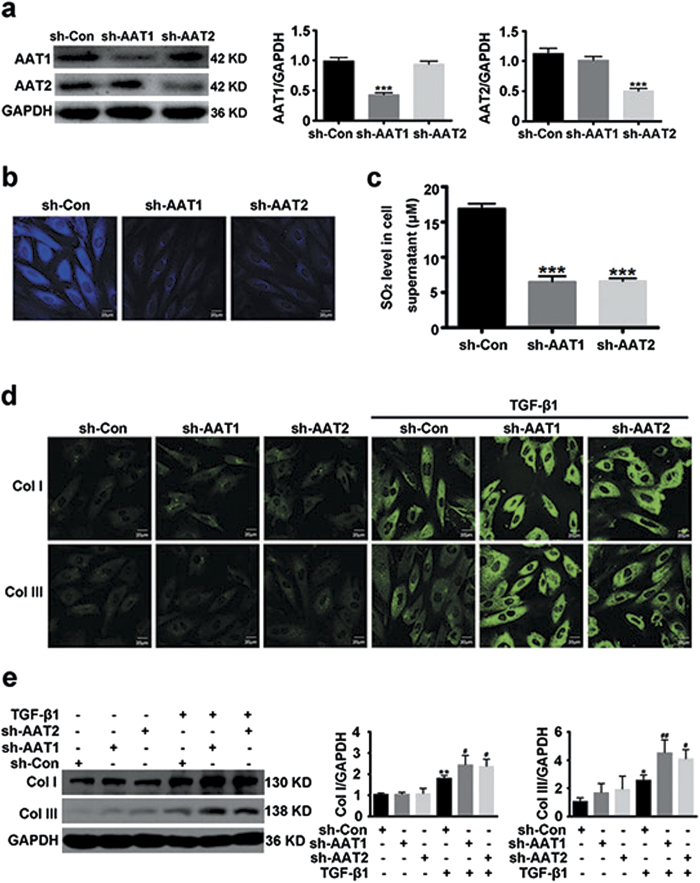
Endogenous SO_2_ knockdown exacerbated TGF-β1-induced VSMC collagen remodeling. (**a**) Protein expression of AAT1 and AAT2 in VSMCs transfected with 2 μg of control shRNA (sh-Con), AAT1 shRNA (sh-AAT1) or AAT2 shRNA (sh-AAT2) for 48 h. (**b**) Representative fluorescent staining (blue) of endogenous SO_2_ in VSMCs transfected with Con, AAT1 or AAT2 shRNA for 48 h. Scale bar, 20 μm. (**c**) SO_2_ content in supernatant from VSMCs transfected with Con, AAT1 or AAT2 shRNA for 48 h. (**d**) Collagen I and III expression in VSMCs by confocal images. VSMCs in coverslips were transfected with Con, AAT1 or AAT2 shRNA before TGF-β1 (10 ng/ml) stimulation for 24 h. Scale bar, 20 μm. (**e**) Representative Western blot and quantification of collagen I and III in VSMCs transfected with Con, AAT1 or AAT2 shRNA before TGF-β1 treatment. ***P < 0.001, **P < 0.01 or *P < 0.05 compared with Con shRNA, ^##^*P* < 0.01 or ^#^*P* < 0.05 compared with Con shRNA + TGF-β1 group (ANOVA). Data are represented as mean ± SD (n = 5).

To further investigate the potential causal role of endogenous SO_2_ in vascular collagen remodeling, we used shRNA to knock down AAT1 or AAT2. Specific knockdown of AAT1 or AAT2 was verified at protein level by Western blot analysis (Fig. 2a). In agreement, transfection with AAT1 shRNA or AAT2 shRNA significantly decreased endogenous SO_2_ in VSMCs, as well as the SO_2_ level in the supernatant (Fig. 2b,c). As compared with control shRNA, endogenous SO_2_ silencing greatly exacerbated TGF-β1-induced collagen I and III expression in VSMCs (Fig. 2d,e). These data probably supported a significant role of endogenous SO_2_ in the regulation of collagen remodeling in VSMCs.

### Endogenous sulfur dioxide likely inhibited the TGF-β1-induced collagen remodeling in VSMCs via suppressing collagen synthesis

**Figure 3:**
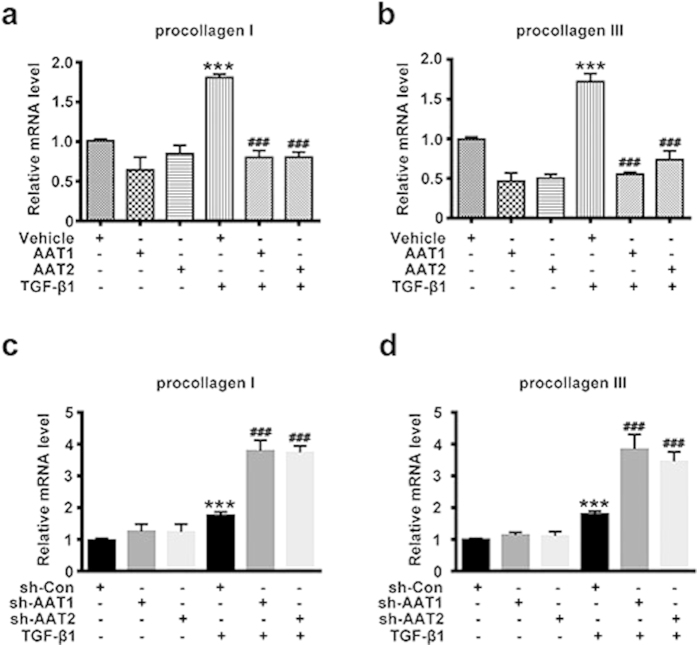
Endogenous SO_2_ regulated TGF-β1-induced VSMC collagen remodeling through inhibiting collagen synthesis. (**a,b**) Procollagen I (**a**) and III (**b**) mRNA expression in VSMCs by real-time PCR. VSMCs were transfected with AAT1, AAT2 or vehicle plasmid before TGF-β1 treatment for 24 h. ***P < 0.001 compared with vehicle, ^###^*P* < 0.001 compared with vehicle + TGF-β1 group (ANOVA). (**c,d**) Procollagen I (**c**) and III (**d**) mRNA expression in VSMCs by real-time PCR. VSMCs were transfected with Con, AAT1 or AAT2 shRNA before TGF-β1 treatment for 24 h. ***P < 0.001 compared with Con shRNA, ^###^*P* < 0.001 compared with Con shRNA + TGF-β1 group (ANOVA). Data are represented as mean ± SD (n = 5).

Collagen synthesis is one major link in collagen expression regulation, mainly reflected in the transcription and translation of procollagen gene. Real-time quantitative PCR (RT-PCR) analysis showed that TGF-β1 stimulation increased the mRNA levels of procollagen I and III, while AAT1 or AAT2 overexpression could significantly inhibit the TGF-β1-induced mRNA expression of procollagen I and III (Fig. 3a,b). In contrast, AAT1 or AAT2 knockdown further exacerbated it (Fig. 3c,d). These results indicated that endogenous SO_2_ likely inhibited the collagen remodeling in VSMCs via inhibiting collagen synthesis.

### Endogenous sulfur dioxide possibly inhibited the TGF-β1-induced collagen remodeling in VSMCs via promoting collagen degradation

**Figure 4:**
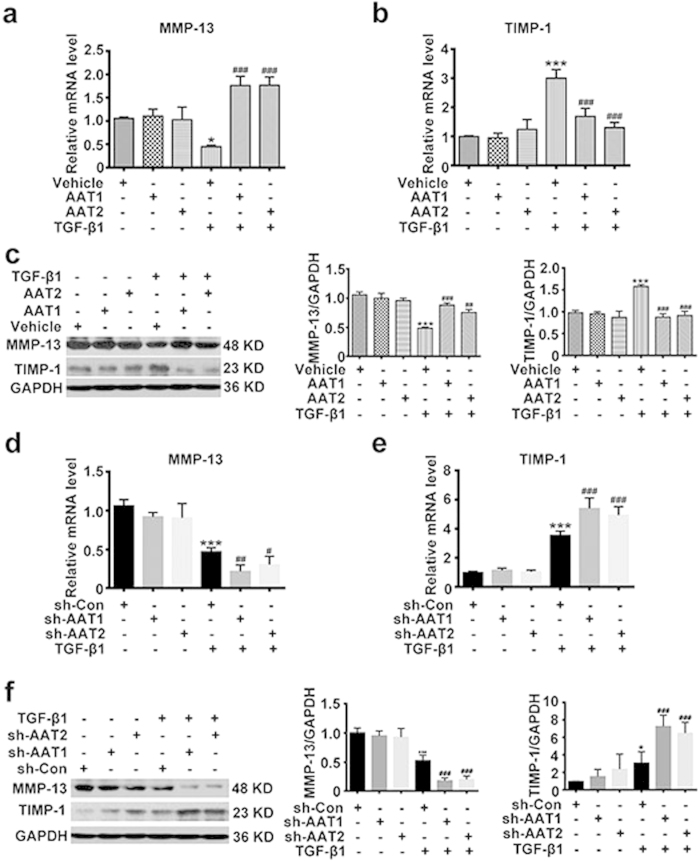
Endogenous SO_2_ regulated TGF-β1-induced VSMC collagen remodeling through promoting collagen degradation. (**a,b**) MMP-13 (**a**) and TIMP-1 (**b**) mRNA expression in VSMCs by real-time PCR. VSMCs were transfected with AAT1, AAT2 or vehicle plasmid before TGF-β1 treatment for 24 h. *P < 0.05 or ***P < 0.001 compared with vehicle, ^###^*P* < 0.001 compared with vehicle + TGF-β1 group (ANOVA). (**c**) Representative Western blot and quantification of MMP-13 and TIMP-1 in VSMCs transfected with AAT1, AAT2 or vehicle plasmid before TGF-β1 treatment for 24 h. ***P < 0.001 compared with vehicle, ^###^*P* < 0.001 or ^##^*P* < 0.01 compared with vehicle + TGF-β1 group (ANOVA). (**d,e**) MMP-13 (**d**) and TIMP-1 (**e**) mRNA expression in VSMCs by real-time PCR. VSMCs were transfected with Con, AAT1 or AAT2 shRNA before TGF-β1 treatment for 24 h. ***P < 0.001 compared with vehicle, ^#^*P* < 0.05, ^##^*P* < 0.01 or ^###^*P* < 0.001 compared with Con shRNA + TGF-β1 group (ANOVA). (**f**) Representative Western blot and quantification of MMP-13 and TIMP-1 in VSMCs transfected with Con, AAT1 or AAT2 shRNA before TGF-β1 treatment for 24 h. ***P < 0.001 or *P < 0.05 compared with Con shRNA, ^###^*P* < 0.001 compared with Con shRNA + TGF-β1 group (ANOVA). Data are represented as mean ± SD (n = 5).

Collagen degradation is the other major link in collagen expression regulation. The key molecules regulating the collagen degradation in VSMCs are matrix metalloproteinase-13 (MMP-13) promoting collagen degradation, and tissue inhibitor of metalloproteinase-1 (TIMP-1) suppressing collagen degradation. RT-PCR and Western blot analysis showed that TGF-β1 downregulated the MMP-13 mRNA and protein levels in VSMCs, whereas increased the TIMP-1 mRNA and protein levels (Fig. 4a–c). AAT1 or AAT2 overexpression in VSMCs could obviously elevate the TGF-β1-downregulated MMP-13 mRNA and protein levels, and suppress the TGF-β1-upregulated TIMP-1 mRNA and protein levels (Fig. 4a–c). In contrast, AAT1 or AAT2 knockdown could further inhibit the TGF-β1-downregulated mRNA and protein expressions of MMP-13, and further promote the TGF-β1-upregulated mRNA and protein expressions of TIMP-1 (Fig. 4d–f). These results suggested that endogenous SO_2_ possibly inhibited the collagen remodeling in VSMCs via promoting collagen degradation.

### Endogenous sulfur dioxide might inhibit the Smad2/3 signaling pathway during TGF-β1-induced collagen remodeling in VSMCs

**Figure 5:**
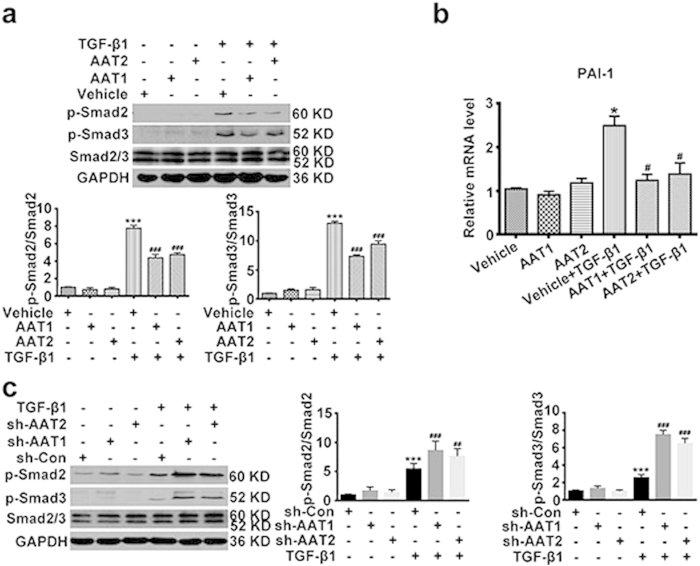
Endogenous SO_2_ inhibited TGF-β1-induced Smad2/3 phosphorylation in VSMCs. (**a**) p-Smad2 and p-Smad3 protein expression in VSMCs transfected with AAT1, AAT2 or vehicle plasmid before TGF-β1 treatment for 1 h. ***P < 0.001 compared with vehicle, ^###^*P* < 0.001 compared with vehicle + TGF-β1 group (ANOVA). (**b**) PAI-1 mRNA expression in VSMCs transfected with AAT1, AAT2 or vehicle plasmid before TGF-β1 stimulation for 8 h. *P < 0.05 compared with vehicle, ^#^*P* < 0.05 compared with vehicle + TGF-β1 group (ANOVA). (**c**) p-Smad2 and p-Smad3 protein expression in VSMCs transfected with Con, AAT1 or AAT2 shRNA before TGF-β1 treatment for 1 h. ***P < 0.001 compared with Con shRNA, ^###^*P* < 0.001 or ^##^*P* < 0.01 compared with Con shRNA + TGF-β1 group (ANOVA). Data are represented as mean ± SD (n = 4–5).

Considering TGF-β/Smad2/3 signal is the key pathway in regulating collagen remodeling, next we observed whether this signaling pathway was involved in the regulation of endogenous SO_2_ on collagen remodeling in VSMCs. Western blot analysis showed that TGF-β1 promoted the phosphorylation of Smad2 and Smad3 as compared with control, while AAT1 or AAT2 overexpression could markedly inhibit the TGF-β1-induced phosphorylation of Smad2/3 (Fig. 5a). To examine if the expression of gene downstream of Smad2/3 was influenced by AAT overexpression, we detected the expression of plasminogen activator inhibitor-1 (PAI-1) using RT-PCR. The data showed that AAT1 or AAT2 overexpression could significantly reduce the TGF-β1-upregulated mRNA expression of PAI-1 (Fig. 5b). On the contrary, AAT1 or AAT2 deficiency dramatically enhanced the TGF-β1-induced phosphorylation of Smad2/3 (Fig. 5c). These data indicated that Smad2/3 signaling pathway might be involved in the modulation of endogenous SO_2_ on collagen remodeling in VSMCs.

### Endogenous sulfur dioxide probably inhibited TβRI phosphorylation during TGF-β1-induced collagen remodeling in VSMCs

**Figure 6:**
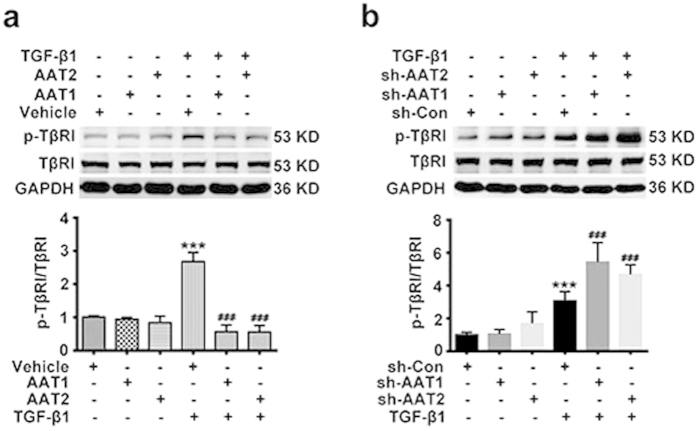
Endogenous SO_2_ inhibited TGF-β1-induced TβRI phosphorylation in VSMCs. (**a**) p-TβRI and TβRI protein expression in VSMCs transfected with AAT1, AAT2 or vehicle plasmid before TGF-β1 treatment for 1 h. ***P < 0.001 compared with vehicle, ^###^*P* < 0.001 compared with vehicle + TGF-β1 group (ANOVA). (**b**) p-TβRI and TβRI protein expression in VSMCs transfected with Con, AAT1 or AAT2 shRNA before TGF-β1 treatment for 1 h. ***P < 0.001 compared with Con shRNA, ^###^*P* < 0.001 compared with Con shRNA + TGF-β1 group (ANOVA). Data are represented as mean ± SD (n = 5).

TGF-β1 binds to TβRII, which phosphorylates TβRI. Then the phosphorylated TβRI triggers Smad2 and Smad3 activation. Therefore, we tested whether endogenous SO_2_ could interfere with phosphorylation of TβRI. Interestingly, TGF-β1 promoted TβRI phosphorylation in VSMCs, while AAT1 or AAT2 overexpression indeed downregulated the TGF-β1-induced TβRI phosphorylation (Fig. 6a). In contrast, AAT1 or AAT2 silencing aggravated the TGF-β1-induced TβRI phosphorylation (Fig. 6b). These results suggested that endogenous SO_2_ might inactivate the TGF-β/Smad2/3 signaling pathway during collagen remodeling of VSMCs through inhibiting TβRI phosphorylation.

### Endogenous sulfur dioxide deficiency promoted collagen remodeling mediated by TGF-β1/Smad signaling pathway in VSMCs

**Figure 7:**
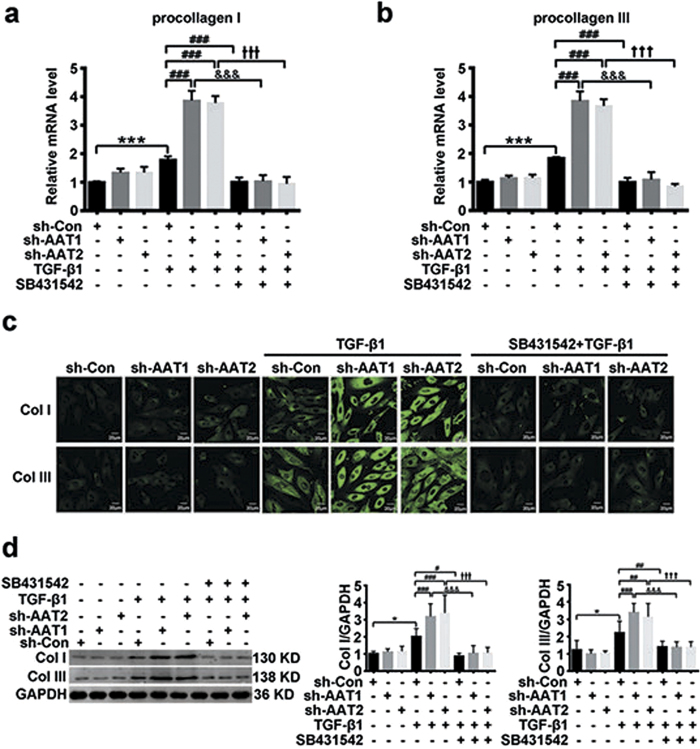
The activation of TGF-β1/Smad signaling pathway mediated endogenous SO_2_ deficiency exacerbating collagen remodeling in VSMCs. (**a,b**) Procollagen I (**a**) and III (**b**) mRNA expression in VSMCs by real-time PCR. VSMCs transfected with Con, AAT1 or AAT2 shRNA were pretreated with SB431542 (5 μmol/L) for 1 h, and then stimulated with TGF-β1 for 24 h. (**c**) Collagen I and III expression in VSMCs by confocal images. VSMCs in coverslips transfected with Con, AAT1 or AAT2 shRNA were pretreated with SB431542 (5 μmol/L) for 1 h, and then stimulated with TGF-β1 for 24 h. Scale bar, 20 μm. (**d**) Representative Western blot and quantification of collagen I and III in VSMCs. VSMCs transfected with Con, AAT1 or AAT2 shRNA were pretreated with SB431542 (5 μmol/L) for 1 h, and then stimulated with TGF-β1 for 24 h. ***P < 0.001 or *P < 0.05 compared with Con shRNA, ^###^*P* < 0.001, ^##^*P* < 0.01 or ^#^*P* < 0.05 compared with Con shRNA + TGF-β1 group, ^&&&^*P* < 0.001 compared with AAT1 shRNA + TGF-β1 group, and ^+++^*P* < 0.001 compared with AAT2 shRNA + TGF-β1 group (ANOVA). Data are represented as mean ± SD (n = 5).

To further explore whether TGF-β1/Smad pathway mediated the regulatory effect of endogenous SO_2_ on collagen remodeling in VSMCs, we applied SB431542, an inhibitor of TGF-β1/Smad signaling pathway, to VSMCs transfected with AAT1 or AAT2 shRNA. RT-PCR showed SB431542 almost completely abolished the AAT1- or AAT2-silencing induction in mRNA expression of procollagen I and III (Fig. 7a,b). Moreover, enhanced collagen I and III protein levels evoked by AAT1 or AAT2 knockdown were also greatly repressed by SB431542 (Fig. 7c,d), suggesting endogenous SO_2_ likely inhibited collagen remodeling at least in part by blocking the activation of TGF-β1/Smad signaling pathway.

### Ectopically expressing AAT abolished AAT deficiency-exacerbated collagen deposition in TGF-β1-stimulatd VSMCs

**Figure 8:**
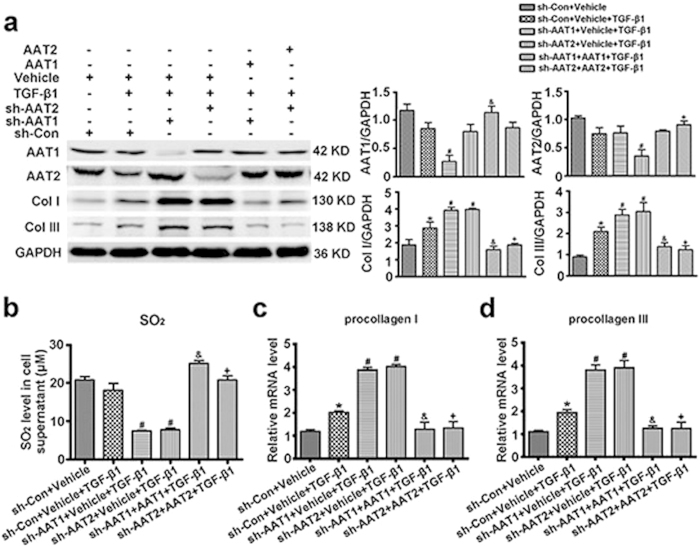
Ectopically expressing AAT abolished AAT deficiency-aggravated collagen remodeling in TGF-β1-treated VSMCs. (**a**) Representative Western blot and quantification of AAT1, AAT2, collagen I and III in VSMCs. VSMCs were co-transfected with Con shRNA and vehicle, AAT1 shRNA and vehicle, AAT2 shRNA and vehicle, AAT1 shRNA and AAT1, or AAT2 shRNA and AAT2 for 48 h, and then stimulated with TGF-β1 for 24 h. (**b**) SO_2_ level in cell supernatant detected by HPLC-FD. (**c,d**) Procollagen I (**c**) and III (**d**) mRNA expression in VSMCs by real-time PCR. VSMCs were co-transfected with Con shRNA and vehicle, AAT1 shRNA and vehicle, AAT2 shRNA and vehicle, AAT1 shRNA and AAT1, or AAT2 shRNA and AAT2 for 48 h, and then stimulated with TGF-β1 for 24 h. *P < 0.05 compared with Con shRNA + vehicle, ^#^*P* < 0.05 compared with Con shRNA + vehicle + TGF-β1 group, ^&^*P* < 0.05 compared with AAT1 shRNA + vehicle + TGF-β1 group, and ^+^*P* < 0.05 compared with AAT2 shRNA + vehicle + TGF-β1 group (ANOVA). Data are represented as mean ± SD (n = 4–5).

In order to exclude the collateral effects that were often produced by shRNA constructs, we ectopically expressed AAT1 or AAT2 in AAT1 or AAT2 deficiency VSMCs, respectively. Western blot analysis showed that transfecting with AAT1 plasmid increased the inhibited protein expression of AAT1 in AAT1-scilencing VSMCs, and transfecting with AAT2 plasmid normalized AAT2 expression in AAT2-knocked down cells (Fig. 8a). Moreover, ectopic AAT1 or AAT2 expression significantly upregulated the inhibited SO_2_ level in AAT deficient VSMCs (Fig. 8b). Of note, AAT1 or AAT2 overexpression substantially abolished the excessive deposition of collagen I and III induced by AAT1 or AAT2 knocked-down in the TGF-β1-treated VSMCs (Fig. 8a). And the enhanced mRNA levels of procollagen I and III by AAT1 or AAT2 deficiency were also retarded by ectopic AAT1 or AAT2 expression (Fig. 8c,d). These data verified that the effects produced by AAT shRNA constructs on collagen remodeling resulted from AAT deficiency.

### Sulfur dioxide but not pyruvate protected against AAT deficiency-induced collagen remodeling in TGF-β1-treated VSMCs

**Figure 9:**
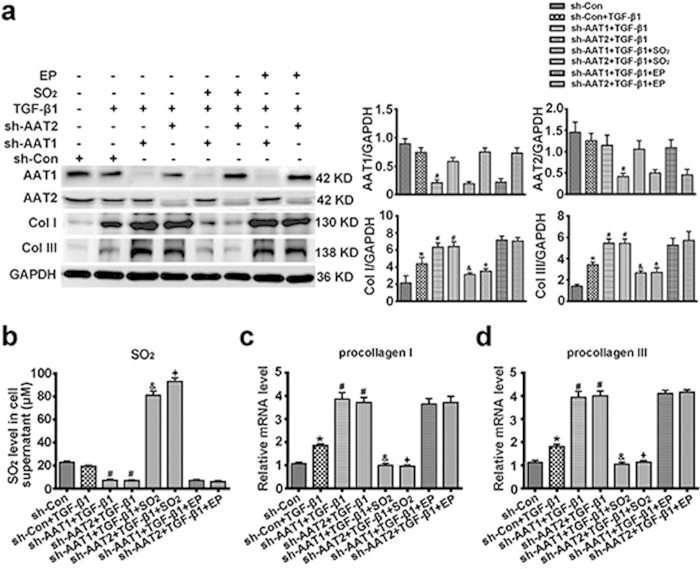
SO_2_ derivatives but not ethyl pyruvate abolished AAT deficiency-promoted collagen remodeling in TGF-β1-treated VSMCs. (**a**) Representative Western blot and quantification of AAT1, AAT2, collagen I and III in VSMCs. VSMCs were transfected with Con shRNA, AAT1 shRNA or AAT2 shRNA for 48 h, pretreated with SO_2_ derivatives (NaHSO_3_/Na_2_SO_3_, 100 μM) or ethyl pyruvate (EP, 100 μM) for 1 h, and then stimulated with TGF-β1 for 24 h. (**b**) SO_2_ level in cell supernatant detected by HPLC-FD. (**c,d**) Procollagen I (**c**) and III (**d**) mRNA expression in VSMCs by real-time PCR. VSMCs were transfected with Con shRNA, AAT1 shRNA or AAT2 shRNA for 48 h, pretreated with SO_2_ derivatives (NaHSO_3_/Na_2_SO_3_, 100 μM) or ethyl pyruvate (EP, 100 μM) for 1 h, and then stimulated with TGF-β1 for 24 h. *P < 0.05 compared with Con shRNA, ^#^*P* < 0.05 compared with Con shRNA + TGF-β1 group, ^&^*P* < 0.05 compared with AAT1 shRNA + TGF-β1 group, and ^+^*P* < 0.05 compared with AAT2 shRNA + TGF-β1 group (ANOVA). Data are represented as mean ± SD (n = 4–5).

Since SO_2_ and pyruvate were generated equimolar from L-cysteine catalyzed by AAT, we next observed the effects of 100 μM SO_2_ derivatives (Na_2_SO_3_/NaHSO_3_, 3:1 mole ratios) and 100 μM ethyl pyruvate on the collagen remodeling induced by AAT deficiency. HPLC-FD analysis showed that SO_2_ derivatives at 100 μM significantly upregulated the inhibited SO_2_ level by AAT1 or AAT2 deficiency, while ethyl pyruvate did not impact endogenous SO_2_ content (Fig. 9b). Western blot analysis demonstrated that 100 μM SO_2_ derivatives could markedly inhibit AAT silencing-aggravated collagen I and III deposition in TGF-β1-treatd VSMCs, while no inhibition was observed at 100 μM ethyl pyruvate (Fig. 9a). Furthermore, 100 μM SO_2_ derivatives but not 100 μM ethyl pyruvate reduced excessive collagen synthesis in AAT1- or AAT2-knocked down plus TGF-β1-treated VSMCs (Fig. 9c,d). These data suggested that SO_2_ but not pyruvate exerted a crucially protective role in AAT deficiency promoted-collagen remodeling in TGF-β1-treated VSMCs.

## Discussion

With the increasing understanding about the function of hydrogen sulfide as gaseous signaling molecule in cardiovascular system, more and more attention has been paid to the physiological and pathophysiological functions of SO_2_, another important gasotransmitter in cardiovascular regulation. Recent studies have found that SO_2_ can be endogenously generated from sulfur-containing amino acid metabolism in human body^7^. L-cysteine can be oxidized into L-cysteine sulfinate by cysteine dioxygenase, the latter can be transformed into β-sulfinylpyruvate by AAT, then spontaneously decomposes to pyruvate and SO_2_^8^. A part of SO_2_ *in vivo* can quickly combine with water to form sulphite, while other exists in gaseous form. Previous Studies found that exogenous SO_2_ donor inhibited the hypoxic pulmonary vascular remodeling^9^. In addition, Liu *et al.* reported that SO_2_ suppressed aortic VSMCs proliferation^15^. However, it’s still unclear about whether endogenous SO_2_ regulates collagen remodeling in VSMCs, and its underlying mechanisms. Therefore, we overexpressed SO_2_ generating enzyme, AAT in VSMCs or knocked down AAT with shRNA, demonstrating endogenous SO_2_ inhibited the TGF-β1-induced excessive collagen deposition in VSMCs and its possible mechanisms.

Maintenance of extracellular matrix (ECM) is one of the major functions of VSMCs. Disturbance of ECM homeostasis leads to excessive ECM deposition, much of which is produced by VSMCs, is a common consequence of cardiovascular diseases such as atherosclerosis, hypertension, and restenosis after coronary angioplasty^16^,^17^,^18^. Most of ECM proteins within vascular walls are collagen I and III^18^. Therefore, searching for endogenous molecules regulating collagen remodeling in VSMCs, can help to protect from the occurrence and development of cardiovascular disease. SO_2_, a gaseous signaling molecule, can be endogenously generated in many cells, including VSMCs^19^. To explore the effect of endogenous SO_2_ on collagen remodeling in VSMCs, we used immunofluorescence staining and Western blot to detect the protein expression of collagen I and III in VSMCs. The data showed that endogenous SO_2_ significantly inhibited the TGF-β1-induced collagen I and III protein levels.

Collagen remodeling contains two major links, namely excessively increased collagen synthesis and/or reduced collagen degradation. Collagen synthesis reflects in the transcription and translation of procollagen gene. So next we detected the effect of endogenous SO_2_ on collagen synthesis. We found that endogenous SO_2_ markedly suppressed the TGF-β1-induced procollagen I and III mRNA levels, suggesting that endogenous SO_2_ could inhibit collagen synthesis in VSMCs.

The balance between degradation of ECM is guaranteed by MMPs and TIMPs^20^,^21^. MMPs consist of a family of Zn-dependent endopeptidases that degrade ECM and basement membrane. They take part in tissue remodeling, cell infiltration and tumor invasion. For example, MMP-2, MMP-9 and MMP-13 are reported to have pivotal roles in vascular remodeling in several disease states^20^,^21^,^22^. MMP-2 mainly degrades type-IV, V, VII, X and XI collagen, gelatin, fibronectin, laminin and elastin. MMP-9 mainly degrades type-IV, V, VII, X collagen, gelatin, elastin, laminin, proteoglycan, fibronectin and entacin. Of note, MMP-13 degrades type-I, II, III, IV, IX, X and XIV collagen, fibronectin, tenascin-C, proteoglycan and gelatin^22^. MMPs activation can be suppressed by their endogenous inhibitors, the TIMPs. Four types of TIMPs have been found, of which TIMP-1 is the important inhibitor of MMP-1, -3, -9 and -13^23^. Therefore, in this study we detected the effect of endogenous SO_2_ on MMP-13 and TIMP-1 mRNA and protein expressions in VSMCs. We found that TGF-β1 downregulated the mRNA and protein expressions of MMP-13, and upregulated the mRNA and protein levels of TIMP-1. Endogenous SO_2_ could dramatically increase the TGF-β1-downregulated MMP-13 level, and decrease the TGF-β1-upregulated TIMP-1 level, suggesting endogenous SO_2_ could promote collagen degradation.

TGF-β1 is a potent profibrotic factor that is involved in vascular fibrosis^24^,^25^,^26^. Previous study reported that TGF-β1 could stimulate collagen synthesis^4^,^27^,^28^, and inhibit collagen degradation^3^,^29^,^30^. Classic TGF signaling pathway is realized by Smad family members^3^,^4^,^12^. Activated TGF-β1 combines to TβRII, which phosphorylates TβRI. Then the phosphorylated TβRI triggers Smad2 and Smad3 phosphorylation. The phosphorylated Smad2/3 forms a complex with Smad4, translocate from cytoplasm into the nucleus and activate the transcription of procollagen^13^,^14^. Previous studies have showed that TGF-β1 activated collagen remodeling in VSMCs mainly through TGF-β/Smad signaling pathway^3^,^4^,^31^. TGF-β1 could significantly increase the collagen protein expression in VSMCs through Smad2/3 signaling pathway^4^,^16^. Our present study showed that endogenous SO_2_ markedly inhibited the TGF-β1-induced phosphorylation of Smad2 and Smad3 in VSMCs. In order to further explore how endogenous SO_2_ blocked the Smad signaling pathway, we detected the phosphorylation level of TβRI. The data indicated that endogenous SO_2_ suppressed the phosphorylation of TβRI. And SB431542, an inhibitor of TGF-β1/Smad signaling pathway, could significantly abolish the promoting effect of endogenous SO_2_ deficiency on TGF-β1-induced collagen remodeling in VSMCs. These results suggested that endogenous SO_2_ alleviated the collagen remodeling in VSMCs at least in part by inhibiting TGF-β/Smad signaling pathway.

The limitation of the present study involved the employment of exclusively one shRNA construct targeting either AAT1 or AAT2, and compared to only one control for all experiments. Considering the collateral effects that were often produced by such constructs, we used ectopically expressing AAT in knocked-down cells. The data showed that ectopic AAT1 or AAT2 expression upregulated the repressed SO_2_ level in AAT knocked down-VSMCs. As a consequence, AAT1 or AAT2 overexpression abolished AAT silencing-induced collagen I and III deposition in TGF-β1-treated VSMCs possibly via reducing collagen synthesis. These results supported the assumption that AAT1 and AAT2 shRNA used in the present study targeted AAT1 and AAT2 genes, respectively. AAT shRNA-induced exacerbation of collagen remodeling might result from AAT deficiency, other than the off-target effects.

Endogenous SO_2_ was produced from L-cysteine in a two-step reaction, in which the later step was catalyzed by AAT and resulted in equimolar pyruvate as well^7^,^8^,^32^. Previous studies showed that pyruvate could modulate TGF-β signaling^33^,^34^. However, whether the effects of AAT manipulation on collagen remodeling were caused by SO_2_ or pyruvate had not been elucidated. Here, we added exogenous SO_2_ derivatives or pyruvate in AAT-knocked down VSMCs. Considering the instability of pyruvate, ethyl pyruvate, a simple aliphatic ester derived from pyruvic acid, was widely implicated in scientific research instead of pyruvate^34^,^35^. Since SO_2_ and pyruvate were generated equimolar from L-cysteine catalyzed by AAT^7^,^8^,^32^, VSMCs were pretreated with 100 μM SO_2_ derivatives or 100 μM ethyl pyruvate in the present study. The results showed that SO_2_ derivatives at 100 μM significantly inhibited AAT silencing-exacerbated collagen I and III deposition in TGF-β1-treated VSMCs, while no inhibition was observed at 100 μM ethyl pyruvate. These data demonstrated that SO_2_ generated by AAT but not pyruvate played a crucially protective role against collagen remodeling in AAT knocked-down VSMCs with TGF-β1 stimulation.

In conclusion, we discovered an inhibitory effect of endogenous SO_2_ on TGF-β1-induced collagen remodeling in VSMCs via suppressing collagen synthesis and promoting collagen degradation. The underlying mechanism might involve inhibiting the TβRI phosphorylation by endogenous SO_2_ to block the Smad2/3 signaling pathway activation. These findings suggest that endogenous SO_2_ may be a promising therapeutic target for vascular collagen remodeling related cardiovascular diseases such as hypertension.

## Methods

### Cell culture

Rat A7r5 VSMCs were obtained from the American Type Culture Collection (Manassas, VA, USA). Cells were cultured in Dulbecco’s modified Eagle’s medium (DMEM) containing 10% FBS, 2 mmol/l glutamine and 20 mmol/l HEPES (pH 7.4) in an atmosphere of 5% CO_2_ at 37 °C.

### Overexpression of AAT1 or AAT2 in VSMCs

The cDNA fragment encoding the full-length rat AAT1 (NM_012571.2) or AAT2 (NM_013177.2) was amplified by PCR and inserted into the pIRES2 vector, and the resultant plasmid AAT1 or AAT2 was verified by DNA sequencing. A7r5 VSMCs were then transfected with AAT1, AAT2 or vehicle plasmid using JetPEI reagent according to the manufacturer’s instructions (Polyplus Transfection, Illkirch, France).

### Knockdown of AAT1 or AAT2 in VSMCs

Rat AAT1 shRNA and AAT2 shRNA were both from OriGene Technologies (Rockville, MD, USA). A7r5 VSMCs were seeded in 6-well plates to 60–80% confluence. AAT1 shRNA, AAT2 shRNA and control shRNA were respectively transfected into A7r5 VSMCs using the JetPEI reagent according to the manufacturer’s instructions (Polyplus Transfection, Illkirch, France).

### Determination of mRNA expressions of procollagen I and III, MMP-13 and TIMP-1 by quantitative real-time polymerase chain reaction

Total RNA was extracted using the Trizol reagent and transcribed into cDNA using oligo (dT) primer and M-MLV reverse transcriptase. Quantitative real-time polymerase chain reaction (RT-PCR) was performed on an ABI PRISM 7300 instrument (Applied Biosystems, Foster, CA, USA). The amplification conditions for the cDNA were: denaturing at 94 °C for 30 s, annealing and polymerizing at 60 °C for 1 min for 40 cycles. Samples and standards were determined in triplicate. TaqMan probes were modified by 5′-FAM and 3′-TAMRA.

Sequences of the primers and probes were: for procollagen I, forward, 5′-CTTGTTGCTGAGGGCAACAG-3′, reverse, 5′-GCAGGCGAGATGGCTTATTC-3′, Taqman probe, 5′-ATTCACCTACACTGTCCTTGTCGATGGCTG-3′; for procollagen III, forward, 5′-GAAAAAACCCTGCTCGGAATT-3′, reverse, 5′-ATCCATCTTGCAGCCTTGGT-3′, Taqman probe, 5′-AGAGACCTGAAATTCTGCCACCCTGAACTC-3′; for MMP-13, forward, 5′- CTTCTGGCACACGCTTTTCC-3′, reverse, 5′-GCTCATGGGCAGCAACAATA -3′, Taqman probe, 5′-CCTGGACCAAACCTTGGCGG -3′; for TIMP-1, forward, 5′-AGCCCTGCTCAGCAAAAGG-3′, reverse, 5′- CTGTCCACAAGCAATGACTGTCA-3′, Taqman probe, 5′- CTTCGTAAAGACCTATAGTGCTGGCTG-3′; for PAI-1, forward, 5′-GGTCAAGATCGAGGTGAACGA-3′, reverse, 5′-GCGGGCTGAGACTAGAATGG, Taqman probe, 5′-CGGCACAGTGGCGTCTTCCTCC-3′; and for β-actin, forward, 5′-ACCCGCGAGTACAACCTTCTT-3′, reverse, 5′-TATCGTCATCCATGGCGAACT-3′, Taqman probe, 5′-CCTCCGTCGCCGGTCCACAC-3′.

### Western blot analysis

A7r5 cells ( 5 × 10^4^) were incubated in 6-well plates. When cells were grown to 60–70% confluences, they were treated as follows. In the first series, cells were transfected with 2 μg of vehicle, AAT1 or AAT2 plasmid. After 24 h, they were starved in DMEM with 0.5% FBS before TGF-β1 (10 ng/ml) treatment for 24 h or 1 h. In the second series, cells were transfected with 2 μg of control shRNA, AAT1 shRNA or AAT2 shRNA for 24 h. Then they were starved for 24 h followed by TGF-β1 (10 ng/ml) treatment for 24 h or 1 h. In the third series, cells were transfected with control shRNA, AAT1 shRNA or AAT2 shRNA for 24 h, staved for 24 h, treated with SB431542 (5 μmol/L) for 1 h, and then stimulated with TGF-β1 (10 ng/ml) for 24 h.

These samples were harvested and lysed in lysis buffer (0.5 mmol/L EDTA, 10 mmol/L Tris–HCl, pH 7.4, 0.3 mol/L sucrose, and protease inhibitor cocktail) as previously described^15^. Equal amounts of protein were resolved on SDS-PAGE gels and transferred onto nitrocellulose membranes. Non-specific bindings were blocked by incubation in 5% milk blocking buffer. The primary antibodies anti-AAT1 and anti-AAT2 were from Sigma-Aldrich Corporation (St Louis, MO, USA), anti-collagen I and anti-collagen III were from Abcam (Cambridge, MA, USA), anti-MMP-13 and anti-TIMP-1 were from Santa Cruz Biotechnology (Santa Cruz, CA, USA), anti-phospho-Smad2, anti-phospho-Smad3 and anti-Smad2/3 were from Cell Signaling Technology (Danvers, MA, USA), and anti-GAPDH were form Kangcheng (Shanghai, China). After incubation, each primary antibody bound with their respective specific horseradish peroxidase-conjugated secondary antibodies (Santa Cruz Biotechnology) and the bands were visualized using enhanced chemiluminescence detection kit (Thermo Scientific, Rockford, IL, USA). The densitometric analysis of the positive bands was performed using AlphaEaseFC (Alpha Innotech Corporation, San Leandro, CA, USA).

### Collagen I and III expression in VSMCs by immunofluorescence and confocal microscopy

VSMCs were plated on glass coverslips. When cells were grown to 60–70% confluences, they were treated as follows. In the first series, cells were transfected with vehicle, AAT1 or AAT2 plasmid. After 24 h, they were starved in DMEM with 0.5% FBS before TGF-β1 (10 ng/ml) treatment for 24 h. In the second series, cells were transfected with control shRNA, AAT1 shRNA or AAT2 shRNA for 24 h. Then they were starved for 24 h followed by TGF-β1 treatment for 24 h. In the third series, cells were transfected with control shRNA, AAT1 shRNA or AAT2 shRNA for 24 h, staved for 24 h, treated with SB431542 (5 μmol/L) for 1 h, and then stimulated with TGF-β1 for 24 h.

The cells were fixed with 4% paraformaldehyde (0.01 mol/L PBS, pH 6.8), washed with PBS, incubated with rabbit anti-collagen I primary antibody (1:25; Sigma-Aldrich) and rabbit anti-collagen III primary antibody (1:25; Sigma-Aldrich) respectively at 4 °C overnight. Next day, after washing, the coverslips were incubated with secondary antibody (1:100) (Invitrogen, Carlsbad, CA) in the dark for 90 min at room temperature. The slides were viewed using a Fluoview laser scanning confocal microscope (Olympus, Tokyo, Japan). In the negative control group, IgG was used instead of the first antibody.

### Measurement of SO_2_ concentration in VSMCs supernatant by high-performance liquid chromatography with fluorescence detection

VSMCs supernatants were collected for SO_2_ content determination. SO_2_ concentrations were measured using high-performance liquid chromatography with fluorescence detection (HPLC-FD, Agilent 1200 series, Agilent Technologies, Palo Alto, CA, USA)^9^. Briefly, 100 μL of VSMCs supernatant was mixed with 70 μL of 0.212 M sodium borohydride in 0.05 M Tris-HCl (pH 8.5) and incubated at 28 °C for 30 min. The sample was then mixed with 10 μL of 70 mM mBrB in acetonitrile, incubated for 10 min at 42 °C, and then mixed with 40 μL of 1.5 M perchloric acid. Protein precipitate in the mixture was removed by centrifugation at 12400×g for 10 min at 25 °C. The supernatant was immediately neutralized by adding 10 μL of 2 M Tris-HCl (pH 3.0), and centrifuged at 12400×g for 10 min. The neutralized supernatant was used for HPLC-FD. Sulfitebimane was measured by excitation at 392 nm and emission at 479 nm. Quantification was carried out by the standardization of sodium sulfite.

### Measurement of endogenous SO_2_ content in VSMCs by a fluorescent probe

Endogenous SO_2_ in VSMCs was measured using a fluorescent probe (kindly provided by Professor Kun Li, College of Chemistry, Sichuan University, Sichuan, China). The culture supernatant of VSMCs was discarded, and washed with PBS for three times. Then, the cells were stained in the working liquid of fluorescent probe (10 μM) for 1 h at 37 °C, washed with PBS and then fixed with 4% paraformaldehyde for 15 min at room temperature. After washed with PBS, the cells were detected as blue fluorescent by confocal microscopy.

### Phosphorylation of TβRI in VSMCs evaluated by immunoprecipitation

VSMCs were seeded in 100-mm dishes for immunoprecipitation. When cells were grown to 60–70% confluences, they were treated as follows. In the first series, cells were transfected with vehicle, AAT1 or AAT2 plasmid. After 24 h, they were starved in DMEM with 0.5% FBS before TGF-β1 (10 ng/ml) treatment for 1 h. In the second series, cells were transfected with control shRNA, AAT1 shRNA or AAT2 shRNA for 24 h. Then they were starved for 24 h followed by TGF-β1 treatment for 1 h.

The cells were then harvested with the antibody against TβRI (Santa Cruz Biotechnology, catalog sc-398) before immunoprecipitation with protein A/G agarose beads (Thermo Fisher Scientific, Waltham, MA, USA)^20^,^36^. The precipitated proteins were resolved by 10% SDS-PAGE and then immunoblotted with antibody against phosphoserine (Abcam, Cambridge, MA, USA)^20^.

### Statistical analysis

Results are presented as mean ± SD. Statistical comparisons were performed with PRISM5 software (GraphPad). Comparisons among more than 2 groups involved one-way ANOVA followed by the Student-Newman-Keuls test for post-hoc comparison as appropriate. *P* < 0.05 was considered significant.

## Additional Information

**How to cite this article**: Huang, Y. *et al.* Endogenous sulfur dioxide alleviates collagen remodeling via inhibiting TGF-β/Smad pathway in vascular smooth muscle cells. *Sci. Rep.* **6**, 19503; doi: 10.1038/srep19503 (2016).
